# Bridging physiological and perceptual views of autism by means of sampling-based Bayesian inference

**DOI:** 10.1162/netn_a_00219

**Published:** 2022-02-01

**Authors:** Rodrigo Echeveste, Enzo Ferrante, Diego H. Milone, Inés Samengo

**Affiliations:** Research Institute for Signals, Systems, and Computational Intelligence sinc(i) (FICH-UNL/CONICET), Santa Fe, Argentina; Medical Physics Department and Balseiro Institute (CNEA-UNCUYO/CONICET), Bariloche, Argentina

**Keywords:** Autism, Neural circuits, Inhibitory dysfunction, Hypopriors, Sampling-based inference

## Abstract

Theories for autism spectrum disorder (ASD) have been formulated at different levels, ranging from physiological observations to perceptual and behavioral descriptions. Understanding the physiological underpinnings of perceptual traits in ASD remains a significant challenge in the field. Here we show how a recurrent neural circuit model that was optimized to perform sampling-based inference and displays characteristic features of cortical dynamics can help bridge this gap. The model was able to establish a mechanistic link between two descriptive levels for ASD: a physiological level, in terms of inhibitory dysfunction, neural variability, and oscillations, and a perceptual level, in terms of hypopriors in Bayesian computations. We took two parallel paths—inducing hypopriors in the probabilistic model, and an inhibitory dysfunction in the network model—which lead to consistent results in terms of the represented posteriors, providing support for the view that both descriptions might constitute two sides of the same coin.

## Author Summary

Two different views of autism, one regarding altered probabilistic computations, and one regarding inhibitory dysfunction, are brought together by means of a recurrent neural network model trained to perform sampling-based inference in a visual setting. Moreover, the model captures a variety of experimental observations regarding differences in neural variability and oscillations in subjects with autism. By linking neural connectivity, dynamics, and function, this work contributes to the understanding of the physiological underpinnings of perceptual traits in autism spectrum disorder.

## INTRODUCTION

Autism spectrum disorder (ASD) refers to a complex neurodevelopmental condition involving persistent challenges in social interaction and communicative skills, and restricted/repetitive behaviors (American Psychiatric Association, 2013). While some recent studies suggest that ASD could be detected during the first year of life in some children, early signs seem to be nonspecific, with group differences more robustly found after a child’s first birthday (see Ozonoff, Heung, Byrd, Hansen, & Hertz-Picciotto, 2008, for a review).

Almost two decades ago, John Rubenstein and Michael Merzenich suggested that many of the symptoms related to ASD might reflect an abnormal ratio between excitation and inhibition leading to hyperexcitability of cortical circuits in ASD subjects (Rubenstein & Merzenich, 2003). Since then, a variety of studies have linked reduced inhibitory signaling in the brain with ASD symptoms, either observing how behavior typically associated with ASD emerges in animals when inhibitory pathways are altered, or measuring gamma-aminobutyric acid (GABA) concentration or GABA receptors in several brain regions (see Cellot & Cherubini, 2014, for a detailed review). Further support for this view comes from the fact that ASD patients suffer from epilepsy with a prevalence up to 25 times that of the neurotypical population (Bolton et al., 2011).

Establishing a direct link between ASD and impaired inhibition in specific circuits in humans has not been easy. Indeed, two recent in vivo studies in humans have shown puzzling results (Horder et al., 2018; Robertson, Ratai, & Kanwisher, 2016). In these studies inhibition was assessed both behaviorally (in visual tasks where inhibition is widely believed to play a key role in neurotypical behavior) and by measuring either GABA concentration (Robertson et al., 2016) or number of GABA receptors (Horder et al., 2018) in the brains of ASD and control subjects. Interestingly, while ASD subjects showed a marked deficit in binocular rivalry, characteristic of a disruption in inhibitory signaling, GABA concentrations in the visual cortex were normal (Robertson et al., 2016). However, while GABA concentration was predictive of rivalry dynamics in controls, the same was not true within the ASD population, evidencing a disruption of inhibitory action. Similarly, while ASD subjects show an altered performance in the paradoxical motion perception task (a proxy measure of GABA signaling), GABA receptor availability in the brain of those participants showed no significant difference from controls (Horder et al., 2018). Both studies suggest an impairment in inhibitory signaling that cannot be explained by coarse differences in GABA concentration or receptor availability at the level of brain areas, and that might affect specific circuits instead. To complicate matters further, there is evidence for not only inhibitory but also excitatory dysfunction in ASD, and it has been hypothesized that homeostatic principles might be the reason behind this seemingly contradictory result (Nelson & Valakh, 2015). The idea is that if, for instance, inhibition is reduced, excitatory synapses might be then adjusted to try to compensate for the overall change in neural activity that reduction would ensue. Computational modeling of local cortical circuits expressed in terms of excitation and inhibition might therefore provide a fruitful avenue of research to guide future experiments.

From the point of view of perception in ASD, a variety of theories have been put forward over the past two decades. Highly influential descriptive theories include the weak central coherence theory (Happé & Frith, 2006) and the enhanced perceptual functioning theory (Mottron, Dawson, Soulieres, Hubert, & Burack, 2006). Here we will focus on computational accounts of perception in ASD, and in particular on a Bayesian view of perception (Palmer, Lawson, & Hohwy, 2017). We will later also make connections to another influential computational theory formulated in terms of predictive coding (Van Boxtel & Lu, 2013; Van de Cruys et al., 2014).

Within the Bayesian framework, inference about the external world proceeds by multiplicatively combining preexistent knowledge (expressed in terms of a *prior* probability distribution) and current sensory evidence (represented in terms of a *likelihood* function), to form a *posterior* distribution that encapsulates our belief about the state of the world after having observed a given stimulus (Knill & Richards, 1996). Rather than expressing that belief as a single point estimate of what is most probable, the posterior distribution provides a richer description, naturally incorporating the associated uncertainty that remains after the observation. A growing body of evidence indicates that, at least in some settings, the brain is able to operate with probability distributions in this way to perform approximate Bayesian inference (see Fiser, Berkes, Orbán, & Lengyel, 2010, for a review). In recent years it has been proposed that in ASD subjects these forms of Bayesian computations are carried out abnormally, overweighting sensory evidence with respect to prior information (Palmer et al., 2017; Pellicano & Burr, 2012). Concretely, the authors in Pellicano and Burr (2012) proposed that this is a consequence of chronically attenuated priors (termed *hypopriors*), characterized by broader distributions (i.e., higher uncertainty).

The related theoretical framework of predictive coding proposes that the cortex is organized following a circuit motif where feedback connections from higher to lower order sensory areas signal predictions of lower level responses, while feedforward connections signal errors between predictions and actually observed lower level responses (Rao & Ballard, 1999). Proponents of predictive coding theories have rightfully pointed out that Bayesian theories by themselves (without specifying a concrete implementation) do not offer a mechanistic explanation for ASD perception (Van Boxtel & Lu, 2013), which is key to understand how physiological observations may be linked to perceptual and behavioral traits in ASD subjects. As has been observed by Aitchison and Lengyel (2017), Bayesian inference and predictive coding are not necessarily mutually exclusive: Predictive coding can be seen as a computational motif that can implement several computational goals (one of which is Bayesian inference), while Bayesian inference can be seen as a computational objective that can have several implementations (one of which is predictive coding). Moreover, as noted in the aforementioned review, telling apart the use of a Bayesian predictive coding scheme from a direct variable code in an empirical setting is no trivial matter. Strong transient overshoots at stimulus onset, for instance, which are a typical signature of predictive coding, can also emerge in direct variable coding schemes (Aitchison & Lengyel, 2016; Echeveste, Aitchison, Hennequin, & Lengyel, 2020). Indeed, while weighting predictive errors more strongly by increasing synaptic gains in the motif could explain sensory hypersensitivity in ASD subjects (Palmer et al., 2017), a competing explanation can be provided within a direct variable coding scheme, as we show in the present study. We note however that while predictive coding schemes can incorporate gamma oscillations (Bastos et al., 2012), it is not clear how they would account for the contrast-dependent frequency modulation of these oscillations (Roberts et al., 2013), or the stimulus-dependent modulations of neural variability (Churchland et al., 2010; Orbán, Berkes, Fiser, & Lengyel, 2016).

A popular implementation choice for probabilistic inference is that of probabilistic population codes (PPCs; Ma, Beck, Latham, & Pouget, 2006), where the posterior distribution is encoded in the average rates of a population of neurons. This framework has been used in the past to link inhibitory deficits and Bayesian computations in an artificial neural network model consisting of two feedforward layers followed by a stage of divisive normalization (Rosenberg, Patterson, & Angelaki, 2015). In this work, a probabilistic version of the model was constructed to capture the “oblique effect.” This term describes the fact that neurotypical subjects tend to be more sensitive to cardinal than to oblique orientations in a visual orientation discrimination task (Westheimer & Beard, 1998). Indeed, a modulation of the divisive normalization factor in this model was shown to account for the observed reduction of the oblique effect in ASD subjects (Dickinson, Jones, & Milne, 2014). The standard PPC framework requires constant Fano factors (no variability modulation; Ma et al., 2006), and furthermore feedforward network implementations can only capture mean rate responses, but fail to account for the dynamical properties of neural responses that arise from recurrent connectivity. It is hence unclear in this framework how altered neural variability observed in the ASD population (Haigh, Heeger, Dinstein, Minshew, & Behrmann, 2015; Milne, 2011) and gamma oscillations (van Diessen, Senders, Jansen, Boersma, & Bruining, 2015) would relate to probabilistic computations in these subjects.

Sampling-based theories for probabilistic inference offer an alternative mechanistic implementation for Bayesian inference. Within this framework, neural circuits represent posterior distributions by drawing samples over time from those distributions (Berkes, Orbán, Lengyel, & Fiser, 2011; Haefner, Berkes, & Fiser, 2016). Interestingly, sampling-based models for probabilistic inference have recently begun to establish direct links between cortical dynamics and perception (Echeveste et al., 2020). A neural circuit model of a cortical hypercolumn respecting Dale’s principle and performing fast sampling-based inference in a visual task displayed a suite of features that are typically observed in cortical recordings across species and experimental conditions. The network showed highly variable responses with strong inhibition-dominated transients at stimulus onset, and stimulus-dependent gamma oscillations, as observed in the cortex (Haider, Häusser, & Carandini, 2013; Ray & Maunsell, 2010; Roberts et al., 2013). The model further evidenced stimulus-dependent variability modulations consistent with experimental findings (Roberts et al., 2013). Divisive normalization of mean responses (Carandini & Heeger, 2012) was also shown to emerge in this network as a result of its recurrent dynamics. This is interesting since divisive normalization was precisely the starting point for the probabilistic model in Rosenberg et al. (2015), and in previous work linking uncertainty and neural variability via gain modulation (Hénaff, Boundy-Singer, Meding, Ziemba, & Goris, 2020). The computational and dynamical properties of the network make it a viable candidate to test the link between Bayesian computations and several physiological features observed in ASD such as inhibitory dysfunction, as well as differences in neural variability and oscillations.

In what follows we will first set the basis for this work by recapitulating some of the key findings of Echeveste et al. (2020), relating probabilistic inference, and dynamics in a network model that we will take to describe healthy control subjects. We will then make use of the connection between perception and physiology established by this model and take two parallel routes to explore two different theories for autism: a perceptual theory expressed in terms of hypopriors, and a physiological theory concerning impaired inhibition. The first path will involve modifying the probabilistic model under which perception takes place, and more concretely its prior, and observing the consequences of that choice in terms of the observer’s posteriors. The second path will involve inducing an inhibitory deficit in the neural network whose job is to sample from the corresponding posteriors, and analyzing the effect of that modification in the posteriors represented by the network. We will then compare the results of both approaches to determine to what extent these two seemingly unrelated theories are compatible. Finally, we show that the induced inhibitory deficit in the network model produces changes in the variability and dynamics of the network. We will evaluate these changes in the context of empirical observations in ASD subjects and other theoretical accounts for ASD. These include an increase in neural variability, as well as an increase in the power and frequency of gamma oscillations. The network also becomes hypersensitive to intense stimuli, displaying stronger transient responses at stimulus onset.

## RESULTS

### Bayesian Inference of Visual Features Implemented by a Recurrent E-I Neural Circuit

**Figure 1.**
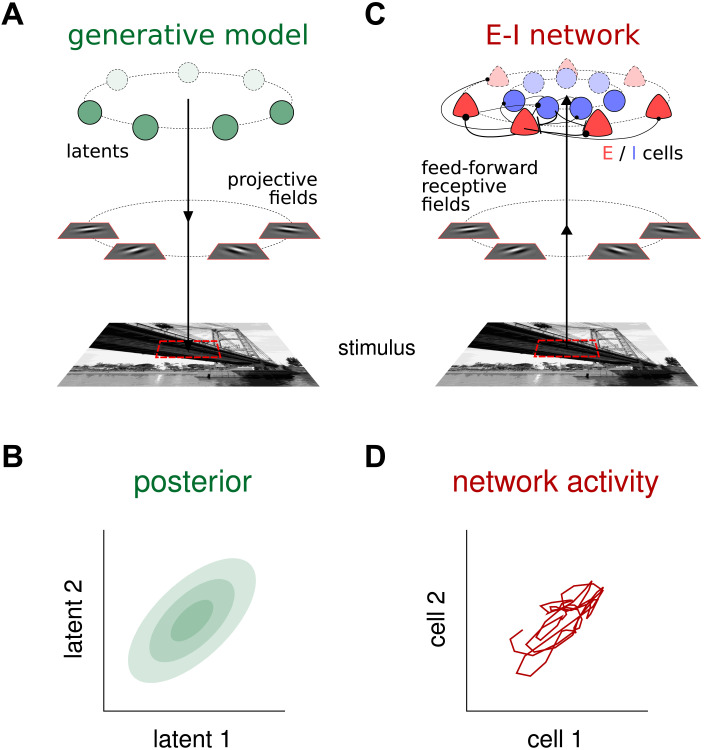
Sketches of the generative model, and a neural circuit implementing sampling-based probabilistic inference under that model. (A) The Gaussian scale mixture (GSM) generative model. Under this model, each image patch is built as a linear combination of local features (projective fields), whose intensities are drawn from a multivariate Gaussian distribution. This linear combination is then further scaled by a global contrast level and subject to noise. The features were in this case a set of localized oriented Gabor filters that differed only in their orientations and were uniformly spread between −90° and 90°. The image serving as stimulus in the figure is for illustration only. Photo credit: Santa Fe Bridge by Enzo Ferrante (https://eferrante.github.io/). (B) A 2D projection of the posterior distribution for a given a visual stimulus as computed by the Bayesian ideal observer under the GSM. (C) The recurrent E–I neural network receives an image patch as an input, which is filtered by feedforward receptive fields matching the projective fields of GSM in panel A. Each latent variable in the GSM is represented by the activity of one E cell in the network. (D) A 2D projection of the neural responses of E cells corresponding the same 2 latent variables shown in panel B. Over time, the network samples from the posterior distribution corresponding to the stimulus it receives.

**Figure 2.**
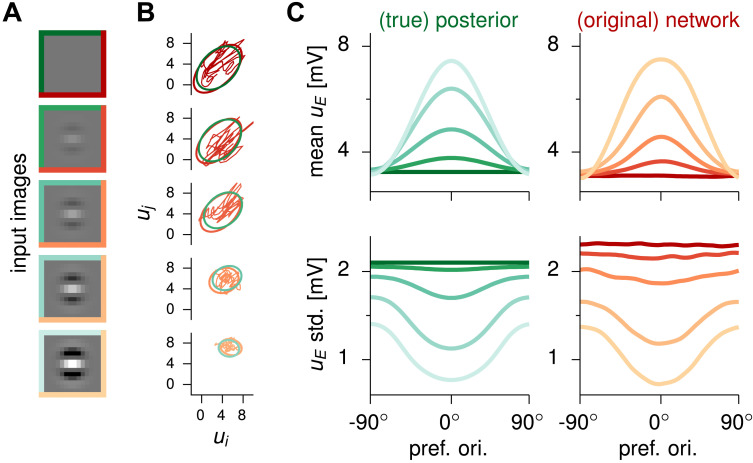
Inference under the GSM and responses in the original network, here representing healthy neurotypical subjects. Replotted from Echeveste et al. (2020). In all panels, shades of green correspond to the ideal observer, while red corresponds to network responses, as in Figure 1. Line colors in panel B indicate different contrast levels, which are the same as stimulus frames in panel A, indicating to which stimulus responses correspond. (A) Stimuli (shade of frame color indicates contrast level, split green, blue, and red indicate that the same stimuli were used as input to the ideal observer and to both neural networks). (B) Covariance ellipses (2 standard deviations) of the ideal observer's posterior distributions (green) and of the networks' corresponding response distributions (red). Red trajectories show sample 500-ms sequences of activities in the networks. As in the sketch of Figure 1, 2D projections corresponding to two representative latent variables / excitatory cells are shown. These two correspond to projective fields / receptive fields at preferred orientations 42° and 16°. (C) Mean (top) and standard deviation (bottom) of latent variable intensities ordered by each latent's orientation, for each stimulus in the training set. Left: from the ideal observer's posterior distribution (green). Right: E cell membrane potentials *u*_*E*_ from the networks' stationary distributions (red). Response moments in panel C were estimated from *n* = 20,000 independent samples (taken 200 ms apart).

The starting point for perceptual inference within the Bayesian framework is a probabilistic model that describes one’s assumptions about how observed stimuli relate to variables of interest in the outside world. This forward model is usually referred to as a *generative model*, and the role of an ideal Bayesian observer is to invert this probabilistic relationship to obtain posterior distributions over those variables of interest given the observed stimulus. The generative model employed here is a Gaussian scale-mixture model (GSM; see Figure 1A and Methods), which has been shown to capture the statistics of natural images at the level of small image patches (Wainwright & Simoncelli, 2000). Importantly, inference under this model had already been shown to explain features of behavior and stationary response distributions in neural data in visual perception (Coen-Cagli, Kohn, & Schwartz, 2015; Orbán et al., 2016; Schwartz, Sejnowski, & Dayan, 2009). Under this version of the GSM, natural image patches are constructed as linear combinations of Gabor filters of different orientations, which are then scaled by a global contrast variable. The goal of the inference process was to estimate the probability distribution of the intensity with which each Gabor filter (each orientation) participated in the observed image. In turn, in order to model cortical neural dynamics, a common recurrent neural network model is employed: the stabilized supralinear network (SSN; see Figure 1B and Methods; Ahmadian, Rubin, & Miller, 2013; Hennequin, Ahmadian, Rubin, Lengyel, & Miller, 2018). Neurons in the network were arranged around a ring, according to their preferred orientation, under the approximation of the visual inference problem being rotationally symmetric (though see Discussion). Moreover, neurons in the network respected Dale’s principle, with two separate populations for excitatory (E) and inhibitory (I) cells. The SSN thus formulated was then optimized using current machine learning methods to approximate a Bayesian ideal observer under the GSM: When the network receives an image patch as its input, it produces samples over time with its neural activity so as to represent the corresponding posterior distribution (Figure 1C–D). Examples of the image patches used to train the network, as well as sample neural trajectories, are presented in Figures 2A and 2B, respectively. After training, posterior distributions sampled by network responses match those prescribed by the ideal observer (see Figure 2C, cf. green and red). Once trained, the SSN model thus establishes a mechanistic link between neural dynamics in terms of an E-I circuit and perception formulated as sampling-based probabilistic inference. In what follows we exploit this link to take two complementary paths: inducing simple perturbations to the GSM to induce hypopriors, and to the SSN to induce an inhibitory dysfunction.

### Perturbing the Generative Model: The Effect of Hypopriors

**Figure 3.**
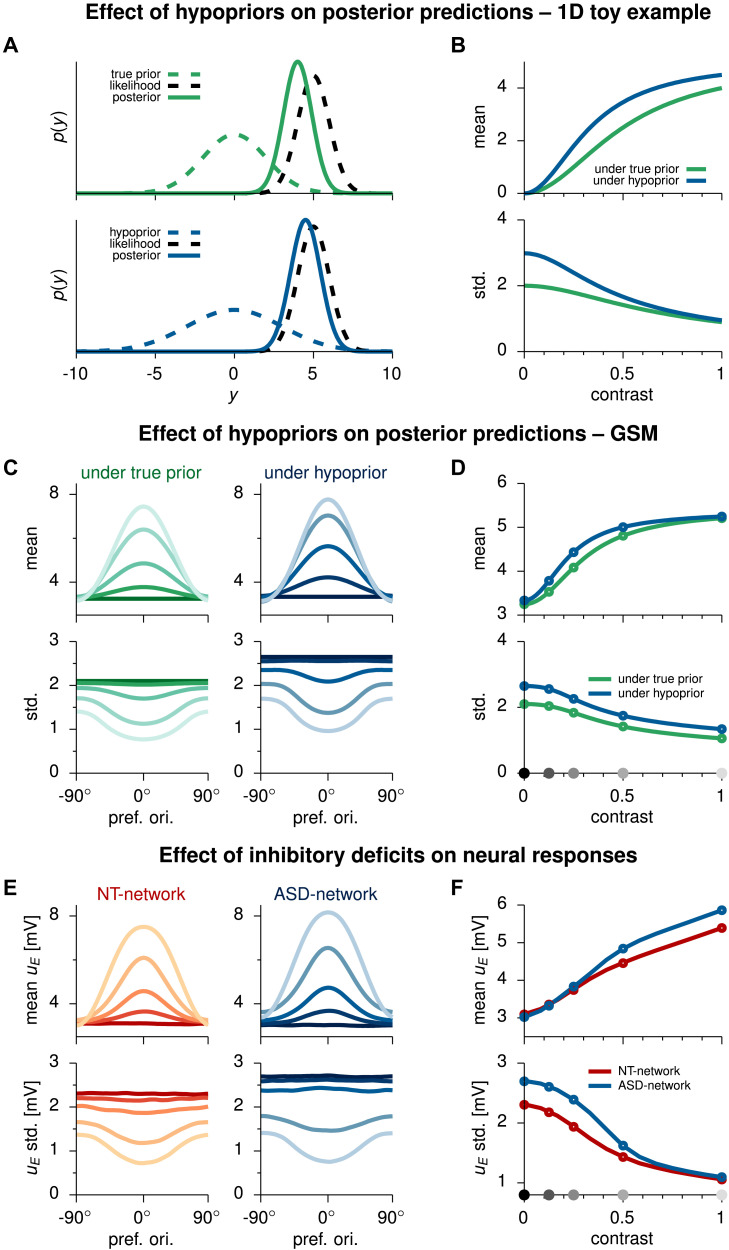
Hypopriors and impaired inhibition. (A, B) Effect of hypopriors on posterior predictions for a 1D toy example. Priors, likelihoods, and posteriors are all Gaussian. A contrast variable regulating the likelihood precision plays the role of the perceptual reliability of stimuli. Two example inference cases are presented: under the true (well-calibrated) prior (dashed, green) and under a wider hypoprior (dashed, blue). (A) The prior (dashed, color) and likelihood (dashed, black) are multiplicatively combined according to Bayes’ rule to form the posterior (continuous, color). (B) Posterior mean (top plot) and standard deviation (bottom plot) under the true prior (green) and the hypoprior (blue), as a function of contrast (likelihood precision). (C, D) Effect of hypopriors on posterior predictions for the full multivariate GSM model. (C) Mean (top plots) and standard deviation (bottom plots) of latent variable intensities ordered by each latent’s orientation, for each stimulus in Figure 2. Left: for the well-calibrated ideal observer’s posterior distribution (green). Right: under a hypoprior (blue). (D) Posterior mean (top) and standard deviation (bottom), averaged across all latent variables, under the true prior (green) and the hypoprior (blue), as a function of contrast. (E, F) Effect of impaired inhibition on network responses. (E) Mean (top) and standard deviation (bottom) of latent variable intensities ordered by each latent’s orientation, for each stimulus in the training set. E cell membrane potentials *u*_E_ from the stationary response distributions for the NT-network (left, red), and for the ASD-network (right, blue). (F) Mean (top) and standard deviation (bottom) of neural responses, averaged across all cells, for the NT-network (red) and the ASD-network (blue), as a function of contrast. Circles, and gray dots on x-axis of panels D and F indicate training contrast levels.

To illustrate and generate intuitions on the effect of hypopriors, we begin by employing a simplified one-dimensional toy example (Methods). Let us assume the “true” prior, correctly describing the statistics of the world concerning a particular inference process, is a zero-mean Gaussian. Let us further assume for this toy example that the likelihood is also a Gaussian function whose precision is modulated by a contrast variable that expresses the degree of reliability of the sensory stimulus. If we vary the stimulus contrast we can compute a posterior distribution for each stimulus under this true prior (Figure 3A–B, in green). If however we were to employ a hypoprior, that is, a prior with a higher variance, we would obtain posterior distributions that overweight sensory evidence, in the sense that they more closely resemble the likelihood function (in both mean and variance) than they should. This in turn results in a higher posterior mean and in higher uncertainty about the estimate (Figure 3B, cf. green and blue lines).

Let us now turn to the GSM. Also in this case, a global contrast variable regulates the reliability of the stimulus. However, in contrast to the one-dimensional toy example presented before, inference in this case takes place in a higher dimensional space. We again modify the prior distribution to induce a hypoprior. We do so in the simplest possible way, by scaling the prior covariance matrix by a constant factor larger than 1.0 (Methods). In Figure 3C we compare the posterior distributions calculated under the true prior (in green) with those computed under the hypoprior (in blue). As expected, we again find that hypopriors result in overweighting of sensory stimuli, with higher posterior means and higher uncertainty about the estimates (Figure 3D, cf. green and blue lines), consistent with the postulates of Pellicano and Burr (2012).

### Perturbing the Network: The Effect of Inhibitory Deficits

We now turn our attention to the network model. In what follows we will refer to the original SSN, presented in Figure 2, as the *neurotypical* (NT) network. As previously stated, the NT-network was constructed in terms of separate excitatory and inhibitory populations. Here we target inhibitory connections by scaling down their efficacy by a global constant value (Methods). In order to ensure that baseline activity levels are not affected, and following the ideas of Nelson and Valakh (2015), we also scaled excitatory connections globally in a homeostatic fashion (see Supplementary Figure 1 and Methods). We will henceforth refer to the network where inhibitory deficits have been induced as the ASD-network. As we did for the generative model, we then compared the mean and standard deviation of the posterior distributions encoded by both networks in terms of their response samples (Figure 3E–F). Notably, we observed that ASD-network representations of the posteriors also seemed to overweight current sensory information. Indeed, posterior means were higher in the ASD- than in the NT-network (Figure 3F top panel, cf. red and blue lines). In passing, we note that because of the original approximate inference scheme, the scaling of the mean and standard deviation with contrast between the original network and the posterior are similar but not identical. In particular, while mean responses in the generative model saturate at high contrasts, they only decelerate in the network model, without actually saturating. Indeed, responses in this type of network models do not saturate. They either continue to grow or “bounce back” and begin to decrease (Ahmadian et al., 2013). Similarly, a slightly higher standard deviation is observed in the network with respect to the posterior at low contrast, which stems from an underestimation of the variance of neural responses under the Gaussian approximation during training of the network (Echeveste et al., 2020).

Higher uncertainty about the estimates was also found in the network (Figure 3F bottom panel, cf. red and blue lines), just as it happened for the generative model under hypopriors (compare Figure 3, panels D and F). Interestingly, we have reached the same qualitative traits by two very different approaches and following two theories expressed at widely different levels: one perceptual, one physiological.

It is important to note that sampling-based implementations of Bayesian inference establish a direct link between uncertainty and neural variability, since the width of the posterior distribution is directly related to the amount of variability. Indeed, we observe that weaker inhibition leads to higher variability in the neural responses of the ASD-network compared with the NT-network (Figure 3F, bottom panel, cf. red and blue lines), as had been suggested in Rubenstein and Merzenich (2003), where the point had been made that a disruption of E-I balance leading to a hyperexcitable cortex would lead to increased cortical “noise.” Indeed, higher neural variability has been experimentally reported in ASD subjects both in EEG (Milne, 2011) and in fMRI (Haigh et al., 2015) studies.

An advantage of employing a neural network model such as the SSN, which shows characteristic features of cortical dynamics, such as gamma oscillations and transient overshoots (including their contrast dependence), is that we can also explore the predictions the model makes for these features, now for the ASD-network.

**Figure 4.**
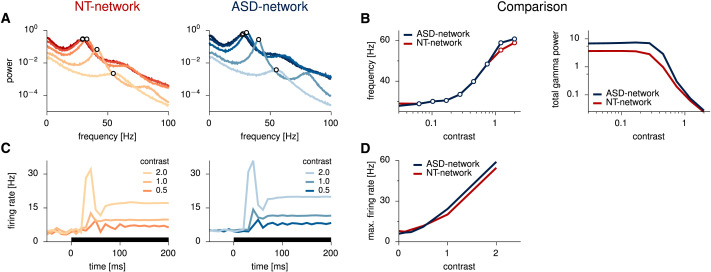
Transient responses and oscillations. (A) LFP power as a function of frequency for stimuli of different contrast levels (same stimuli and colors as in Figure 3) in the NT-network (left), and in the ASD-network (right). Both networks present strong gamma oscillations (see peaks in the gamma band, indicated by empty circles). (B) Comparison of oscillatory behavior in both networks. On the left, the peak gamma frequency is presented as a function of stimulus contrast for both networks. Very minimal differences are observed. On the right, the total power within the gamma band is presented as a function of contrast for both networks. A higher gamma power is observed for the ASD-network at all contrasts, with strong differences at low contrasts. (C) Across-trial average transient responses for stimuli of different contrast levels in the neurotypical network (left) and in the ASD-network (right). Both networks present strong stimulus-dependent transient overshoots. (D) Comparison of overshoot sizes. The maximal firing rate is presented as a function of stimulus contrast for both networks. We observe that the ASD-network presents stronger peak responses at higher contrasts, overreacting to intense stimuli. NT-network results reproduced from Echeveste et al. (2020).

First, we look at gamma oscillations. To that end we computed the power spectrum from the local field potential (LFP), from which we extracted the peak gamma frequency for different contrast levels for both networks (Figure 4A). We note that the overall frequency modulation is very similar in both networks, with slightly higher peak gamma frequency in the ASD-network for high contrast stimuli (cf. Figure 4B, left panel, red and blue). Previous work has reported higher peak gamma frequency in ASD subjects solving a visual task, which was interpreted as a sign of “increased neural inhibition” (Dickinson, Bruyns-Haylett, Smith, Jones, & Milne, 2016). At first glance, this might seem at odds with the starting point for our work where we have weakened inhibitory synapses. It is worth noting however that total inputs (both E and I) result in a balanced recurrent network from a dynamic equilibrium, which may result in higher inhibitory currents, despite weaker inhibitory synapses. This is precisely the case here (see Supplementary Figure 1D). Indeed, it has been known for decades that balanced networks are prone to so-called paradoxical effects (Tsodyks, Skaggs, Sejnowski, & McNaughton, 1997), whereby direct external inhibitory inputs to I cells can actually lead to increased I rates. This also hints at why seemingly contradictory results are often found regarding inhibition in ASD depending on what exactly is chosen as a measure of inhibition.

Interestingly, gamma power is higher for the ASD-network (see sharper gamma peaks in the spectra of Figure 4A, and in Figure 4B, right plot, blue vs. red). An insight into the functional interpretation of this effect can be obtained from analyzing neural responses at zero contrast, representing what is usually termed spontaneous activity in the literature. In sampling-based models, such as this one, spontaneous activity is postulated to encode the prior distribution (Berkes et al., 2011). Indeed, when the stimulus is completely uninformative, as is the case at zero contrast, the posterior matches the prior. The model hence predicts higher gamma power in spontaneous activity, which is in line with previous reports of higher gamma band power in resting-state activity of ASD subjects (van Diessen et al., 2015).

We finally turn our attention to transient responses. We compared the ASD- and NT-networks in terms of their trial-averaged firing rates around stimulus onset (Figure 4C). The model predicts higher maximal firing rates (and not only mean rates) for the ASD-network than for the NT-network at intermediate and high contrasts (cf. Figure 4D, red and blue), indicating that the ASD-network has become hypersensitive to intense stimuli. We note that theories of perception expressed in terms of predictive coding usually interpret peak rates as a measure of surprise, novelty, or unexpectedness (Rao & Ballard, 1999), and indeed a predictive coding account of ASD perceptual traits, including abnormal sensory sensitivity, has been postulated by several authors in the past (Van Boxtel & Lu, 2013; Van de Cruys et al., 2014). Results from the ASD-network, which we here interpret from a Bayesian inference perspective, are then not inconsistent with a predictive coding view of perceptual differences in the ASD population.

## DISCUSSION

Neural network models are increasingly being used as a tool to study how differences in neural architectures may be linked to symptoms in different disorders (Lanillos et al., 2020). In this work we have employed a neural network model of a V1 cortical hypercolumn trained to perform sampling-based probabilistic inference in a visual task to build a mechanistic bridge between descriptions of ASD formulated at two very different levels: a physiological level, in terms of inhibitory dysfunction (Rubenstein & Merzenich, 2003), neural variability (Haigh et al., 2015; Milne, 2011), and gamma oscillations (van Diessen et al., 2015); and a perceptual level, in terms of hypopriors in Bayesian computations (Pellicano & Burr, 2012). In what follows we describe merits of this work, limitations, and open questions.

### Merits

We have taken two parallel paths: in one perturbing the probabilistic generative model in order to induce hypopriors, and in the other perturbing the neural network model to induce an inhibitory dysfunction. We observed that both approaches lead to consistent results in terms of the represented posterior distributions, providing support for the possibility that both views of ASD might actually constitute two sides of the same coin.

Employing a neural network model such as the SSN, which not only performs inference in a perceptual task but also displays characteristic features of cortical dynamics while doing so (Echeveste et al., 2020), allowed us to make further connections between characteristic differences in these dynamics and inhibitory dysfunction in ASD subjects. Stimulus-dependent variability modulations in the network, and concretely the direct link between neural variability and uncertainty established by sampling-based implementations of inference, predicted higher variability in neural responses in the ASD- versus the NT-network. Indeed, increased neural variability has been reported in ASD subjects both in EEG (Milne, 2011) and in fMRI (Haigh et al., 2015) studies. Moreover, transient overshoots, usually interpreted in predictive coding theories to represent novelty, surprise, or unexpectedness (Rao & Ballard, 1999), are present in the network, with higher responses for strong stimuli in the ASD-network versus the NT-network, indicating an oversensitivity to intense stimuli, a feature often reported in children with ASD (Kern et al., 2006).

Furthermore, oscillations in the ASD-network displayed higher gamma band oscillatory power, consistent with observations in resting-state EEG recordings of ASD subjects (van Diessen et al., 2015). Peak gamma frequencies were also higher in the ASD-network for high-contrast stimuli, a fact that has indeed been observed in EEG recordings from subjects performing an orientation discrimination task (Dickinson et al., 2016), and that had been attributed to increased inhibition. We confirmed that, despite having decreased the efficacy of inhibitory synapses in our network, mean inhibitory inputs were indeed actually larger for high-contrast stimuli. This observation is in line with the known fact that balanced E-I networks are prone to “paradoxical effects” regarding inhibition (Tsodyks et al., 1997), where average rates result from a dynamic balance of excitation and inhibition, and might explain apparent contradictions between studies reporting increased/decreased inhibition (Cellot & Cherubini, 2014; Dickinson et al., 2016). These results also highlight the importance of neural network simulations to assist in the interpretation of physiological observations regarding the role of inhibition in cortical recordings.

### Limitations and Open Questions

Training recurrent neural networks with expansive nonlinearities beyond mean responses is currently a challenging and computationally expensive task. These networks are prone to instabilities and current optimization for second-order moments requires either a large number of trials, or matrix-matrix operations that scale as *n*^3^ in the number of neurons (Hennequin & Lengyel, 2016). Indeed, the choice of the simple generative model played a key role in order to make the training problem tractable with currently available optimization techniques, but it imposes some limitations. The GSM produces multivariate Gaussian posteriors (which enabled training the network with currently available second-order moment-matching methods), and was further constructed to be rotationally symmetric (which drastically reduced the number of network parameters to be optimized, as well as the required number of training examples). A model constructed in this way will however not be able to capture features of human behavior in popular tests of visual perception, such as the “oblique effect,” where neurotypical subjects seem to be more sensitive to cardinal orientations (Westheimer & Beard, 1998), an effect that is reduced in ASD subjects (Dickinson et al., 2014). Tackling problems like these in a sampling-based setting will require developing tools to train more flexible networks that can produce richer posterior distributions. It should be noted that these limitations are, however, of a technical nature, and are not inherent to the sampling-based inference framework.

Second, the model employed to explain simple, low-level perceptual computations was constructed in terms of a single V1 hypercolumn, and is hence only able to capture local dynamical features, such as locally generated gamma oscillations. Hypothetically, the ideas presented here can be extended to the representation of other circular variables beyond orientation of visual stimuli, such as head direction in rodents (Skaggs, Knierim, Kudrimoti, & McNaughton, 1995), motor intent in primates (Georgopoulos, Taira, & Lukashin, 1993), physical space in grid cells (McNaughton, Battaglia, Jensen, Moser, & Moser, 2006), or oculomotor control (Seung, 1998). In all these examples, highly specialized brain areas receive assorted inputs that carry a noisy, filtered, and distributed representation of a circular variable. The recurrent activity of the network constitutes a mechanistic implementation of an inference process, which could be potentially executed through a sampling-based Bayesian inference strategy, as explored here. If that were the case, the strong reliance of ASD subjects on the likelihood could also be broadened beyond the realm of sensory processing. Extensions of these ideas are also conceivable to other one-dimensional, yet aperiodic, domains, such as sound pitch (Aronov, Nevers, & Tank, 2017), navigation speed (Kropff, Carmichael, Moser, & Moser, 2015), or elapsed time (Tsao et al., 2018) which, although still fairly narrow in their semantic content, involve some degree of higher level processing. However, as we progress into still higher cognitive functions, the understanding of how context-dependent modulations of cortical dynamics emerge during complex perceptual tasks will likely require models where multiple circuits interact (Simon & Wallace, 2016). In this sense, hierarchical or spatially extended versions of the SSN model employed here may provide adequate substrates to study inference of higher level perceptual tasks where longer range aspects of cortical dynamics, such as gamma synchronization, might emerge.

Third, we have focused on one aspect of probabilistic inference: inferring the state of a set of latent variables under perceptual uncertainty. The study of other aspects of this problem, such as inferring temporal transitions (Sinha et al., 2014), or causal relationships (Noel, Shivkumar, Dokka, Haefner, & Angelaki, 2021), and their link to altered inhibition and neural dynamics, will require the use of different architectures and generative models and constitute worthwhile avenues of future research.

### Closing Remarks

We have shown how recurrent neural networks optimized for sampling-based inference are viable candidates to bridge the gap between Bayesian perceptual theories of ASD and their physiological underpinnings in terms of inhibitory dysfunction, neural variability, and oscillations. We believe these results highlight the potential for the use of the emerging body of function-optimized neural networks (Echeveste et al., 2020; Hennequin, Vogels, & Gerstner, 2014; Orhan & Ma, 2017; Remington, Narain, Hosseini, & Jazayeri, 2018; Song, Yang, & Wang, 2016; Yamins et al., 2014) as models to establish mechanistic links between neural activity and computations in the cortex that go beyond the study of neurotypical perception.

## METHODS

In order to link cortical dynamics and probabilistic computations we modified the parameters of the probabilistic and network models employed in Echeveste et al. (2020). In what follows we describe those changes and refer the reader to the original paper for a more detailed description of the models and of the original model parameters.

### The Generative Model

In this work the Gaussian scale mixture model (GSM; Wainwright & Simoncelli, 2000) is employed as the generative model of natural images (at the level of small patches) under which inference is carried out in the primary visual cortex (V1; Coen-Cagli et al., 2015; Orbán et al., 2016). Under the GSM an image patch **x** is obtained by linearly combining a number of local features (given by the columns of a matrix **A**), which are weighted by a corresponding number of feature coefficients given by **y**, further scaled by a single contrast variable *z*, and finally corrupted by additive white Gaussian noise. This forward generative model can then be summarized in terms of the likelihood function given by

$$x|y,z∼𝒩\left(z\;A\;y,\sigma_{x}^{2}I\right),$$ (1)

together with the priors for the feature coefficients and the contrast variable *z*. Local features were assumed to be drawn from a multivariate Gaussian:

$$y∼𝒩\left(0,C\right),$$ (2)

and the contrast was assumed to be drawn from a gamma prior. To induce hypopriors we modified the overall scale of the prior covariance matrix **C**, by taking **C**_HP_ = *α*_HP_**C**, with *α*_HP_ = 1.5. Other values were explored without qualitative differences (not shown). We note that taking *α*_HP_ > 1 results in wider priors, as required for a hypoprior.

The one-dimensional toy example model of Figure 3A–B, corresponds to a one-dimensional GSM with prior variance *C* = 4, *A* = 10, and $\sigma_{x}^{2}$ = 100. As in the full GSM, we took *α*_HP_ = 1.5.

### Network Dynamics and Architecture

The circuit model consisted of a nonlinear, stochastic network respecting Dale’s principle, with *N*_E_ excitatory and *N*_I_ inhibitory neurons. The evolution of the membrane potential *u*_*i*_ of each neuron *i* in this model is described by Hennequin et al. (2018):

$$\tau_{i}\frac{du_{i}}{dt}=-u_{i}\left(t\right)+h_{i}\left(t\right)+\sum_{j}W_{ij}r_{j}\left(t\right)+\eta_{i}\left(t\right),$$ (3)

where *τ*_*i*_ represents the membrane time constant for neuron *i*, *h*_*i*_ is its feedforward input, and *η*_*i*_ is the process noise (capturing both intrinsic and extrinsic forms of neural variability). **W** is the matrix of recurrent connections, and hence *W*_*ij*_ represents the strength of the synapse connecting neuron *j* to neuron *i*. As previously mentioned, the network is nonlinear, with firing rates

$$r_{i}\left(t\right)=k{\left\lfloor u_{i}\left(t\right)\right\rfloor }^{m}.$$ (4)

Here *k* and *m* represent the scale and exponent of the firing rate nonlinearity (Ahmadian et al., 2013). Given the rotational symmetry of the problem, **W** itself was parametrized to be rotationally symmetric. Neurons in the model are arranged in a ring of pairs of E and I cells according to their preferred orientations (Figure 1C), where *W*_*ij*_ was a smoothly decaying function of the tuning difference between neurons *i* and *j* (see Supplementary Figure 1A, top and second row). The (stimulus-independent) process noise covariance was analogously parametrized (see Supplementary Figure 1A, third row). Following canonical models of V1 simple cells (Dayan & Abbott, 2001), feedforward inputs to the network were computed by applying a linear filter **W**^ff^ to the stimulus (the image patch) followed by a nonlinearity (see Supplementary Figure 1A, bottom row).

The perturbation here employed to induce an inhibitory deficit has a single free parameter *δ*_I_ that scales the inhibitory columns of **W**, $W_{I}^{ASD}$ = (1 − *δ*_I_)$W_{I}^{NT}$ (see Supplementary Figure 1A–B). In order to maintain the baseline level of activity, a second modification is introduced (simulating homeostatic adaption of the excitatory connections), scaling the excitatory columns of **W** by a factor *δ*_E_: $W_{E}^{ASD}$ = (1 − *δ*_E_)$W_{E}^{NT}$. This second factor was found by grid-search minimization of the homeostatic cost

$${𝒞}_{h}=\left|\mu_{s}^{NT}-\mu_{s}^{ASD}\right|,$$ (5)

capturing the change in mean spontaneous activity levels (*μ*_s_) between the original NT- and perturbed ASD-network. This adaptation procedure returns a single *δ*_E_ value for each *δ*_I_ value (Supplementary Figure 1C). We note that excitatory changes via this procedure always resulted smaller than inhibitory ones (cf. to identity line in Supplementary Figure 1C, bottom plot). Network results presented throughout this paper correspond to *δ*_I_ = 0.1, for which *δ*_E_ = 0.076. Numerical experiments were repeated for *δ*_I_ = 0.05 and *δ*_I_ = 0.15 without qualitative differences (not shown).

### Numerical Simulations

Stationary moments of neural responses to a fixed input (Figure 3E) were computed from 20,000 independent samples (200 ms apart) generated by letting neural activity in the network evolve over time via Equation 3 (excluding transients). Power spectra in Figure 4A were obtained from simulated local field potentials (LFPs), computed as the average (across-cell) membrane potential. Gamma peak frequencies in Figure 4B (left) were obtained as the local maximum in the spectrum within the gamma range (20–80 Hz), while total gamma power in Figure 4B (right) was computed as the integral of the spectrum over that same range.

Transient responses displayed in Figure 4C were computed as the mean (across E-cells and trials) firing rates (*n* = 100), which are then further averaged over a 10-ms sliding window. A random delay time (sampled from a truncated Gaussian, with a mean of 45 ms and a standard deviation of 5 ms) was employed for the feedforward input to each pair of E–I cells. These procedures had been put in place to allow for a comparison to experimental data, and are here kept in order to compare the ASD-netowork to replotted results from the original (here NT-) network. Maximal firing rates in Figure 4D were obtained as the peak rates from transient firing rate responses.

## ACKNOWLEDGMENTS

This work was supported by Argentina’s National Scientific and Technical Research Council (CONICET), which covered all researchers’ salaries. We are grateful to Y. Nagai for pointing out this potential avenue of research after discussing previous work.

## CODE AVAILABILITY

The (Python) code to create the ASD-network is provided in bitbucket.org/RSE_1987/inhibitory_dysfunction (Echeveste, 2021). The code for the numerical experiments can be found at bitbucket.org/RSE_1987/ssn_inference_numerical_experiments (Echeveste, 2020).

## SUPPORTING INFORMATION

Supporting information for this article is available at https://doi.org/10.1162/netn_a_00219.

## AUTHOR CONTRIBUTIONS

Rodrigo Echeveste: Conceptualization; Formal analysis; Investigation; Visualization; Writing – original draft. Enzo Ferrante: Conceptualization; Writing – original draft. Diego H. Milone: Conceptualization; Supervision; Writing – original draft. Inés Samengo: Conceptualization; Supervision; Writing – original draft.

## FUNDING INFORMATION

Rodrigo Echeveste, Santa Fe Agency for Science, Technology, and Innovation, Award ID: IO-138-19.

## TECHNICAL TERMS

GABA: Main inhibitory neurotransmitter.
Prior: Probability distribution encapsulating an observer’s knowledge about the latent variables before observing the stimulus.
Likelihood function: Function describing the conditional probability of an observation for each state of the latent variables.
Posterior: Conditional probability over the latent variables after observing a given stimulus.
Hypoprior: A chronically attenuated prior, whose uncertainty is higher than implied by the statistics of stimuli.
Transient overshoot: Excursion in neural responses that exceeds mean responses over a brief period of time after the onset of the stimulus.
Gamma oscillations: Rhythmic patterns of activity with a frequency between 20 and 80 Hz.
Divisive normalization: Process by which the responses of single neurons are divisively modulated by the responses of other neurons.
Latent variable: A variable of interest to which an observer has no direct access and hence needs to infer from an observation of other related variables.

## Supporting Information

https://bitbucket.org/RSE_1987/ssn_inference_numerical_experiments

https://bitbucket.org/RSE_1987/inhibitory_dysfunction
