# Supplementary material for: Bridging physiological and perceptual views of autism by means of sampling-based Bayesian inference
Source: Netw Neurosci. 2022 Feb 1;6(1):196–212. doi: 10.1162/netn_a_00219 (PMC9810278; doi:10.1162/netn_a_00219)
Supplement: Supplementary file 1 [file netn-06-196-s001.pdf]

Echeveste, R., Ferrante, E., Milone, D. H & Samengo, I. (2022). supporting information for "Bridging physiological and perceptual views of autism by means of sampling based Bayesian inference." *Network Neuroscience*, 6(1), 196–212. https://doi.org/10.1162/netn_a_00219

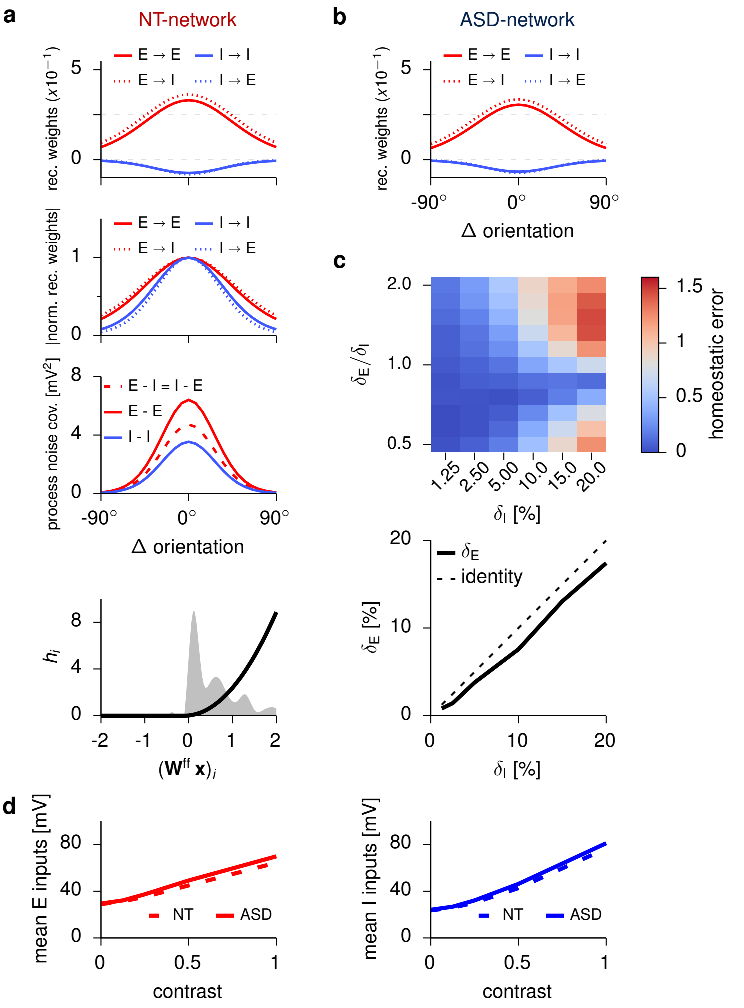

Supplementary Fig. 1 | a, Parameters of the NT-network. Given the symmetry of the problem, both the synaptic weights (top two rows) and the input noise covariance (third row) are given by circulant matrices constructed by the templates presented here. The bottom row depicts the input nonlinearity (in black) and, as a reference, the distribution of inputs across all cells for the training set. **b,** Recurrent weight templates for the perturbed neural network, regarded here as the ASD network. All other parameters are shared with the NT-network **c,** Homeostatic adjustment of excitation. Top: Color plot represents homeostatic error (deviation of baseline activity levels) as a function of the ratio of changes in excitation and inhibition (δ_E/δ_I), for different choices of δ_I. Bottom: For each value of δ_I, the value of δ_E which minimizes the homeostatic error in the top plot is selected via grid search. The dashed line corresponds to the identity function. The ASD network presented in **b**, and employed throughout this work corresponds to the $\delta_I = 10\%$ case. **d,** Average total recurrent excitatory (left) and inhibitory (right) inputs to the ASD-network (full lines) and the NT-network (dashed lines) as a function of contrast.
